# Structural Characterization of Multicomponent Crystals Formed from Diclofenac and Acridines

**DOI:** 10.3390/ma15041518

**Published:** 2022-02-17

**Authors:** Artur Mirocki, Artur Sikorski

**Affiliations:** Faculty of Chemistry, University of Gdańsk, W. Stwosza 63, 80-308 Gdańsk, Poland

**Keywords:** 2-(2-(2,6-dichloroanilino)phenyl)acetic acid, diclofenac, acridine, 6,9-diamino-2-ethoxyacridine, ethacridine, crystal structure, hydrogen bonds, π–π interactions, crystal packing

## Abstract

Multicomponent crystals containing diclofenac and acridine (**1**) and diclofenac and 6,9-diamino-2-ethoxyacridine (**2**) were synthesized and structurally characterized. The single-crystal XRD measurements showed that compound **1** crystallizes in the triclinic *P*-1 space group as a salt cocrystal with one acridinium cation, one diclofenac anion, and one diclofenac molecule in the asymmetric unit, whereas compound **2** crystallizes in the triclinic *P*-1 space group as an ethanol solvate monohydrate salt with one 6,9-diamino-2-ethoxyacridinium cation, one diclofenac anion, one ethanol molecule, and one water molecule in the asymmetric unit. In the crystals of the title compounds, diclofenac and acridines ions and solvent molecules interact via N–H⋯O, O–H⋯O, and C–H⋯O hydrogen bonds, as well as C–H⋯π and π–π interactions, and form heterotetramer bis[⋯cation⋯anion⋯] (**1**) or heterohexamer bis[⋯cation⋯ethanol⋯anion⋯] (**2**). Moreover, in the crystal of compound **1**, acridine cations and diclofenac anions interact via N–H⋯O hydrogen bond, C–H⋯π and π–π interactions to produce blocks, while diclofenac molecules interact via C–Cl⋯π interactions to form columns. In the crystal of compound **2**, the ethacridine cations interact via C–H⋯π and π–π interactions building blocks, while diclofenac anions interact via π–π interactions to form columns.

## 1. Introduction

Non-steroidal anti-inflammatory drugs (NSAIDs) are one of the most used group of drugs due to their broad spectrum of biological activity [1,2]. They are used, inter alia, as analgesic and anti-inflammatory drugs [3,4], can reduce the risk of some cancers [5] and are used in treating Alzheimer’s disease [6,7], as well as many other applications [8,9,10]. One non-steroidal drugs is diclofenac (IUPAC: 2-(2-(2,6-dichloroanilino)phenyl)acetic acid). It is a commonly used drug for degenerative joint disease, various inflammations, such as spondylitis, and is also used after surgery and dysmenorrhea [11,12,13,14]. Moreover, diclofenac is a fast-acting drug and has analgesic and antipyretic activities, used to treat actinic keratosis and may have an antituberculosis effect [15,16,17,18,19,20,21]. Therapeutic doses of diclofenac cause fewer side effects (such as gastrointestinal damage) than aspirin and naproxen, and it is also inhibitor of cyclo-oxygenase [22]. Moreover, diclofenac is detected in water and may adversely affect organisms and thus disturb ecosystem function [23,24,25,26,27].

One of the fields dealing with the design, preparation, and research of new forms of drugs is crystal engineering. Thanks to our understanding of the nature of intermolecular interactions, such as hydrogen bonds [28,29,30], halogen bonds [31,32,33], π–π stacking interactions [34,35,36], and others [37,38,39,40], it is possible to obtain APIs as multicomponent crystals such as salts, cocrystals, salt cocrystals, or their solvates [41,42,43]. From the pharmaceutical point of view, the most interesting are the multicomponent crystals containing more than one Active Pharmaceutical Ingredients (APIs), the physicochemical and pharmacological properties of which may be better than single APIs [44,45,46,47]. A search of the Cambridge Structure Database (CSD version 5.43, update November 2021) [48] shows that there are 76 crystal structures of organic compounds containing diclofenac, including 58 structures of salts, 13 structures of cocrystals and 5 structures of salt cocrystals. However, there are only a few examples showing the possibility of formation crystalline drug–drug system involving diclofenac and aromatic nitrogenous bases [49,50,51,52,53,54,55,56,57].

In the context of obtaining a crystalline drug–drug system, acridine derivatives are very interesting. This is due to fact that acridines can intercalate into DNA which makes them exhibit anticancer, antibacterial, antiprion, and antiviral effects, among others [58,59,60,61,62]. Our previous research shows that acridine and its derivatives may interact with coformer molecules through various intermolecular interactions, such as hydrogen bonds, halogen bonds, π–π stacking interactions, and are able to form good quality crystals that are stable at room temperature [63,64,65,66]. One of the acridine derivatives represent interesting medicinal activity is ethacridine. Ethacridine (6,9-diamino-2-ethoxyacridine) is active against various infections and has antibacterial and anticancer properties, among others [67,68,69,70].

**Scheme 1:**
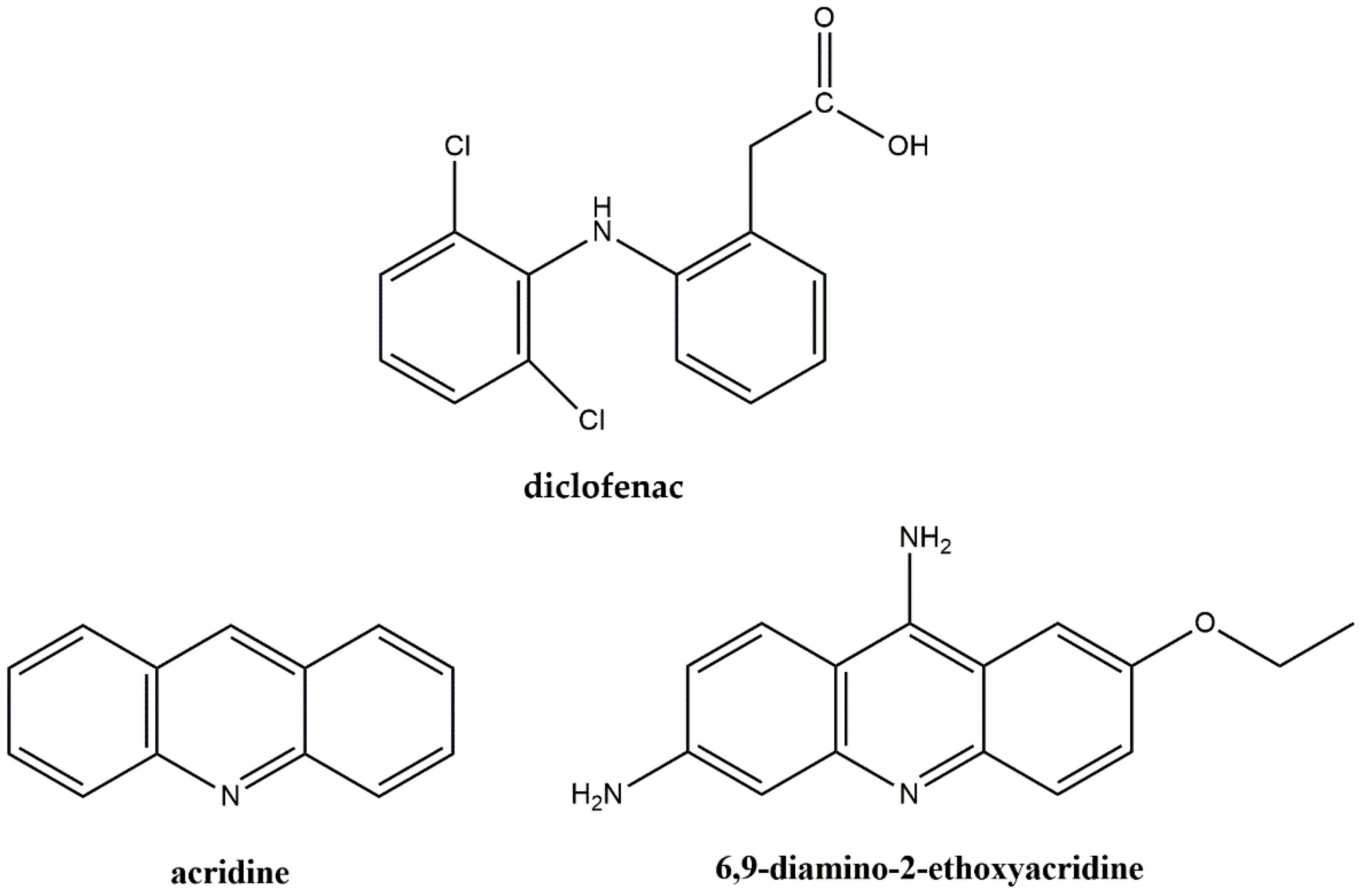
Chemical structures reported in this paper.

With regard to the biological activity of diclofenac and acridinium derivatives, in this article we describe the synthesis and structural characterization of two multicomponent crystals formed from diclofenac and acridine (**1**) and diclofenac and 6,9-diamino-2-ethoxyacridine (**2**) (Scheme 1).

## 2. Materials and Methods

All the chemical compounds were purchased from Sigma-Aldrich (St. Louis, MO, USA) (acridine, 6,9-diamino-2-ethoxyacridine-DL-lactate monohydrate) and Tokyo Chemical Industry TCI (diclofenac) and used without purification. Melting points were determined on a Büchi M-565 (Flawil, Switzerland) capillary apparatus and were uncorrected.

### 2.1. Synthesis of Compounds ***1*** and ***2***

(**1**) Acridine (0.020 g, 0.111 mmol) and diclofenac (0.030 g, 0.111 mmol) were dissolved in 4 mL of an ethanol/water mixture (1:1 *v*/*v*) and heated for 20 min in order to dissolve the sample. The solution was allowed to evaporate for a few days to give yellow crystals of **1** (m.p. = 158.0 °C).

(**2**) 6,9-Diamino-2-ethoxyacridine-DL-lactate monohydrate (0.020 g, 0.0552 mmol) and diclofenac (0.016 g, 0.0552 mmol) were dissolved in 4 mL of an ethanol/water mixture (1:1 *v*/*v*) and heated for 20 min in order to dissolve the sample and evaporate the lactic acid. The solution was allowed to evaporate for a few days to give yellow crystals of **2** (m.p. = 138.9 °C).

### 2.2. X-ray Measurements and Refinements

Good-quality single-crystal specimens of compounds **1** and **2** were selected for X-ray diffraction experiments at T = 295(2) K (Table 1). Diffraction data were collected on an Oxford Diffraction Gemini R ULTRA Ruby CCD diffractometer (Oxfordshire, United Kingdom) with MoKα (λ = 0.71073 Å) radiation. The lattice parameters were obtained by least-squares fit to the optimized setting angles of the reflections collected by means of CrysAlis CCD (ver. 1.171.41.16a) [71]. Data were reduced using CrysAlis RED software (ver. 1.171.41.16a) [71] and applying multi-scan absorption corrections. The structures were solved with direct methods that carried out refinements by full-matrix least-squares on *F*^2^ using the SHELXL-2017/1 program (ver. 2017/1) [72].

All H atoms bound to N/O-atoms were located on a difference Fourier map and refined freely. All H atoms bound to aromatic C atoms were placed geometrically and refined using a riding model with *d*_(C–H)_ = 0.93 Å and U_iso_(H) = 1.2U_eq_(C). All H atoms from the methyl group were positioned geometrically and refined using a riding model, with *d*_(C–H)_ = 0.96 Å and U_iso_(H) = 1.5U_eq_(C). All H atoms from the water molecules were positioned geometrically and refined using a riding model, with *d*_(O–H)_ = 0.85 Å and U_iso_(H) = 1.5U_eq_(O) (DFIX command). All interactions and the Kitaigorodskii type of packing index were calculated using the PLATON program (ver. 181115) [73]. The following programs were used to prepare the molecular graphics: ORTEPII [74], PLUTO-78 [75], and Mercury (ver. 2020.2.0) [76]. Full crystallographic details for title compound have been deposited in the Cambridge Crystallographic Data Center (deposition No. CCDC 2,128,053 and 2,128,054) and they may be obtained from http://www.ccdc.cam.ac.uk, (accessed on 25 January 2022) email: deposit@ccdc.cam.ac.uk, or The Director, CCDC, 12 Union Road, Cambridge, CB2 1EZ, UK.

## 3. Results

### 3.1. Crystal Structure of Compound ***1***

**Figure 1:**
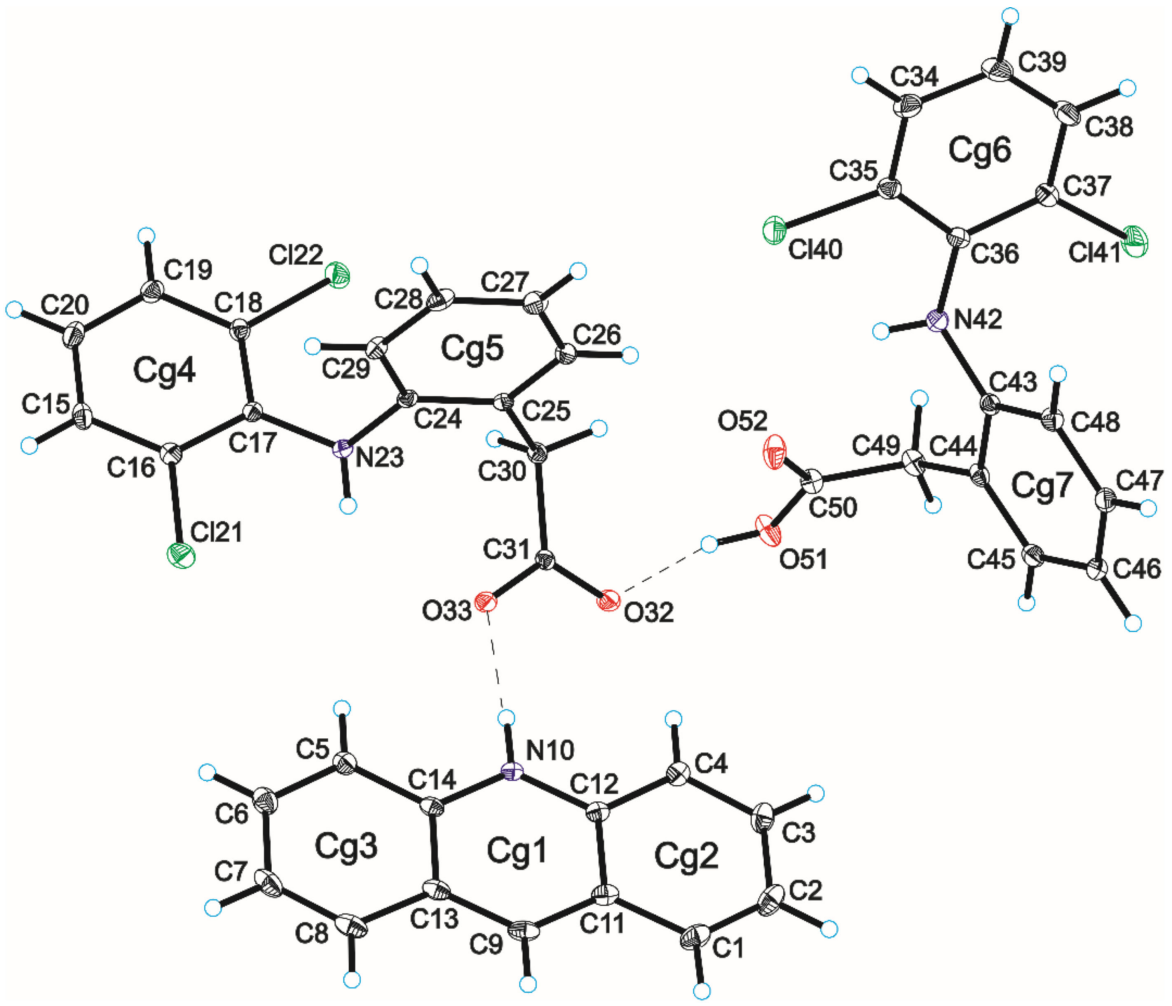
Molecular structure of compound **1** showing the atom-labelling scheme (Cg1, Cg2, Cg3, Cg4, Cg5, Cg6, and Cg7 denote the ring centroids; hydrogen bonds are represented by dashed lines).

The single-crystal X-ray diffraction measurements show that compound **1** crystallizes in the triclinic *P*-1 space group with one acridinium cation, one diclofenac anion and one diclofenac molecule in the asymmetric unit (Figure 1).

**Figure 2:**
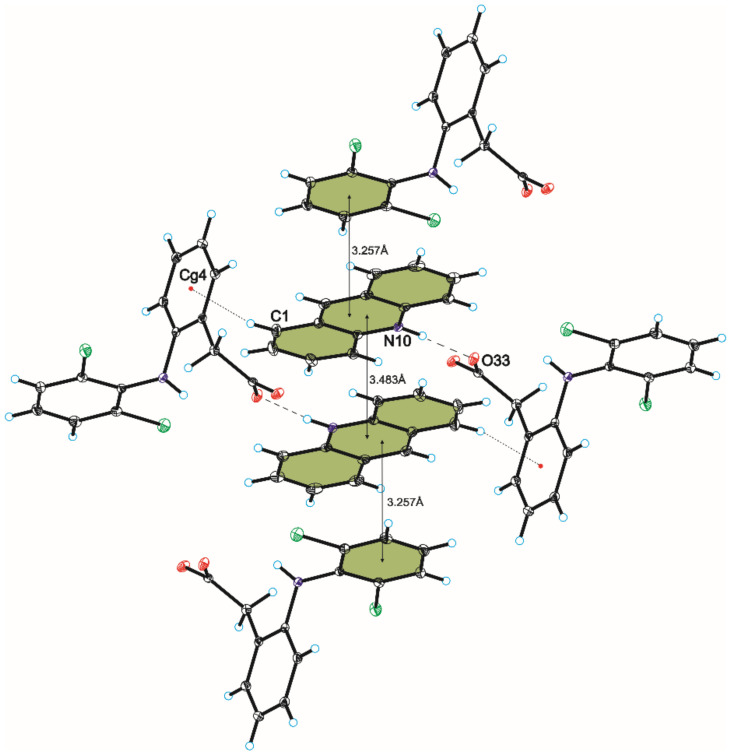
Heterotetramer formed by acridinium and diclofenac ions in compound **1** (hydrogen bonds are represented by dashed lines, whereas C–H⋯π interactions are represented by dotted lines).

The lengths of C–O bonds from carboxylic group in diclofenac anion (1.249 Å and 1.260 Å) indicating a proton transfer occurring between the carboxylic group of diclofenac and acridine. An acridine skeleton is nearly planar, whereas the angle between the planes of aromatic rings is 87.2° and 60.6°, and the angle –C–N–C– are 118.9° and 123.5° in diclofenac anion and molecule, respectively. We also observe intramolecular N–H⋯O hydrogen bonds [*d*(H23⋯O33) = 2.39(2) Å, and ∠(N23–H23⋯O33) = 130 (2)°, and *d*(H42⋯O52) = 2.36 (2) Å, and ∠(N42–H42⋯O52) = 142 (2)°] in both diclofenac anion and molecule (Table 2, Figure 1). In the crystal of **1**, diclofenac anion and molecule are linked via O_(carboxy)-_H⋯O_(carboxy)_ hydrogen bond [*d*(H51⋯O32) = 1.69 (3) Å, and ∠(O51–H51⋯O32) = 164 (3)°] to form a monoanionic dimer (Figure 1). The acridinium cation interact with diclofenac anion through the N_(acridine)_–H⋯O_(carboxy)_ hydrogen bond [*d*(H10⋯O33) = 1.79 (2) Å, and ∠(N10–H10⋯O33) = 170 (2)°], and C_(acridine)_–H⋯π intermolecular interaction [*d*(H1⋯Cg4) = 2.79 Å, and ∠(C1–H1⋯Cg4) = 165°] to form a cyclic heterotetramer stabilized by the weak C_(acridine)_–H⋯O_(carboxy)_ hydrogen bonds between C-4 atom of acridinium cation and an O-atom from carboxylate group of diclofenac anion [*d*(H4⋯O32) = 2.40 Å, and ∠(C4–H4⋯O32) = 160°] (Table 2 and Table 3, Figure 2). In this heterotetramer, the adjacent acridinium cations interact via π–π stacking with centroid⋯centroid distances [*d*(Cg⋯Cg)] ranging from 3.737 (1) Å to 3.841 (1) Å and separation 3.483 Å between the mean planes of the acridine skeleton (Table 4, Figure 2).

**Figure 3:**
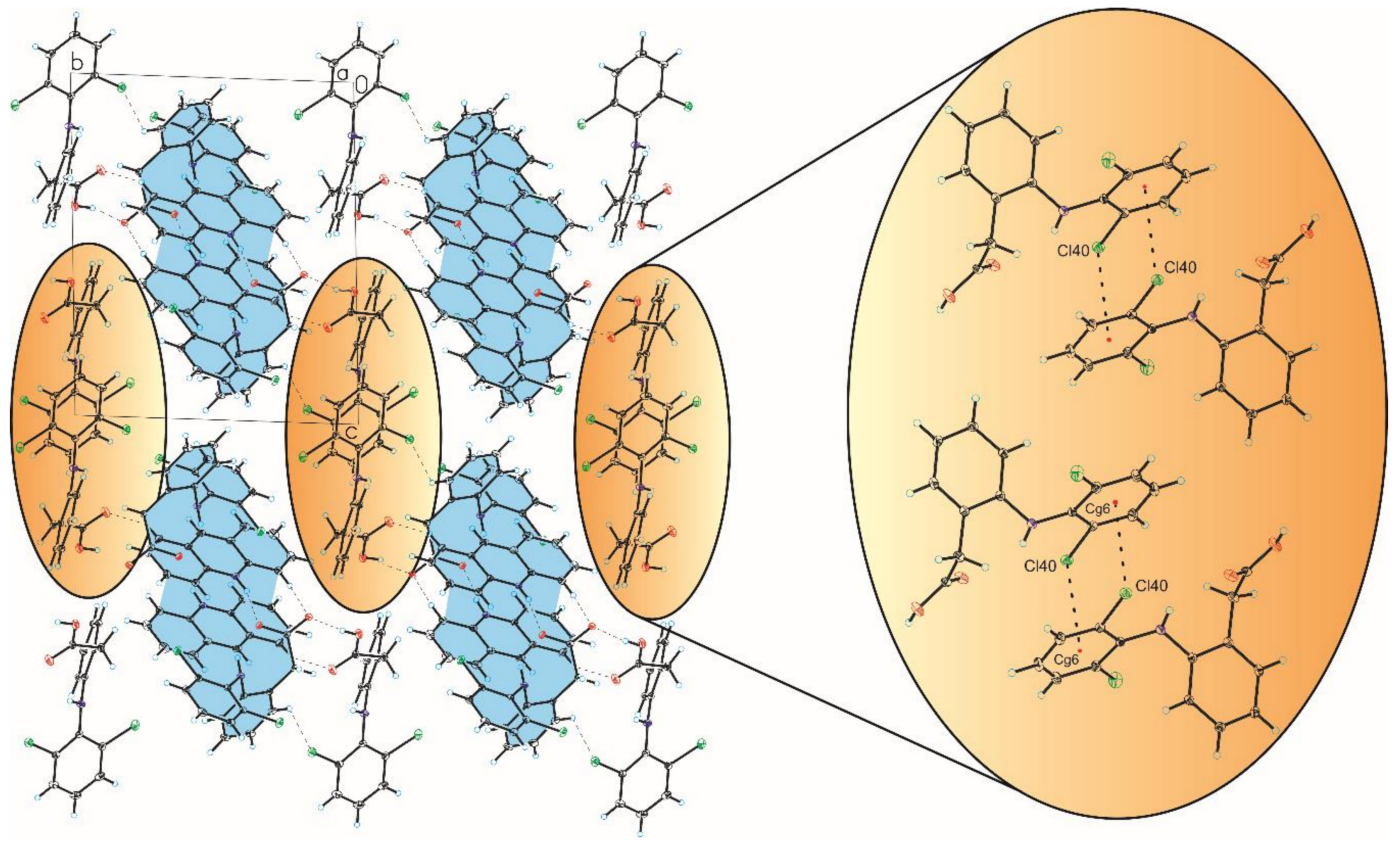
Crystal packing of compound **1** viewed along the *a*-axis (hydrogen bonds are represented by dashed lines, whereas C–Cl⋯π interactions are represented by dotted lines).

Moreover, the neighboring heterotetramers interact via π–π interactions between the acridine skeleton and the aromatic ring of the diclofenac anion (Cg4) with centroid⋯centroid distances ranging from 3.662 (1) Å to 3.917 (1) Å and separation 3.257 Å, to form blocks along the *a*-axis (Table 4, Figure 3—highlighted in blue). The adjacent diclofenac molecules are connected by C–Cl⋯π intermolecular interaction [*d*(Cl40⋯Cg6) = 3.725 (1) Å, and ∠(C35–Cl40⋯Cg6) = 95.86 (8)°] to form columns (Table 3, Figure 3—highlighted in orange). The adjacent blocks are connected to each other through columns by C–Cl⋯π interaction [*d*(Cl21⋯Cg7) = 3.885(1) Å, and ∠(C16–Cl21⋯Cg7) = 127.92 (2)°], and the weak C–H⋯O_(carboxy)_ hydrogen bond [*d*(H45⋯O32) = 2.71 (1) Å, and ∠(C45–H45⋯O32) = 160 (1)°], between the diclofenac anion and molecule, and C_(acridine)_–H⋯π intermolecular interaction [*d*(H7⋯Cg7) = 2.90 Å, and ∠(C7–H7⋯Cg7) = 156°] between the acridine cation and the diclofenac molecule (Table 3, Figure 3).

They are stabilized by the weak C_(acridine)_–H⋯O_(carboxy)_ hydrogen bonds between the C-8 atom of the acridinium cation and an O-atom from the carboxyl group of the diclofenac molecule [*d*(H8⋯O52) = 2.50 Å, and ∠(C8–H8⋯O52) = 150°] to form a 3D framework structure (Figure 3).

### 3.2. Crystal Structure of Compound ***2***

**Figure 4:**
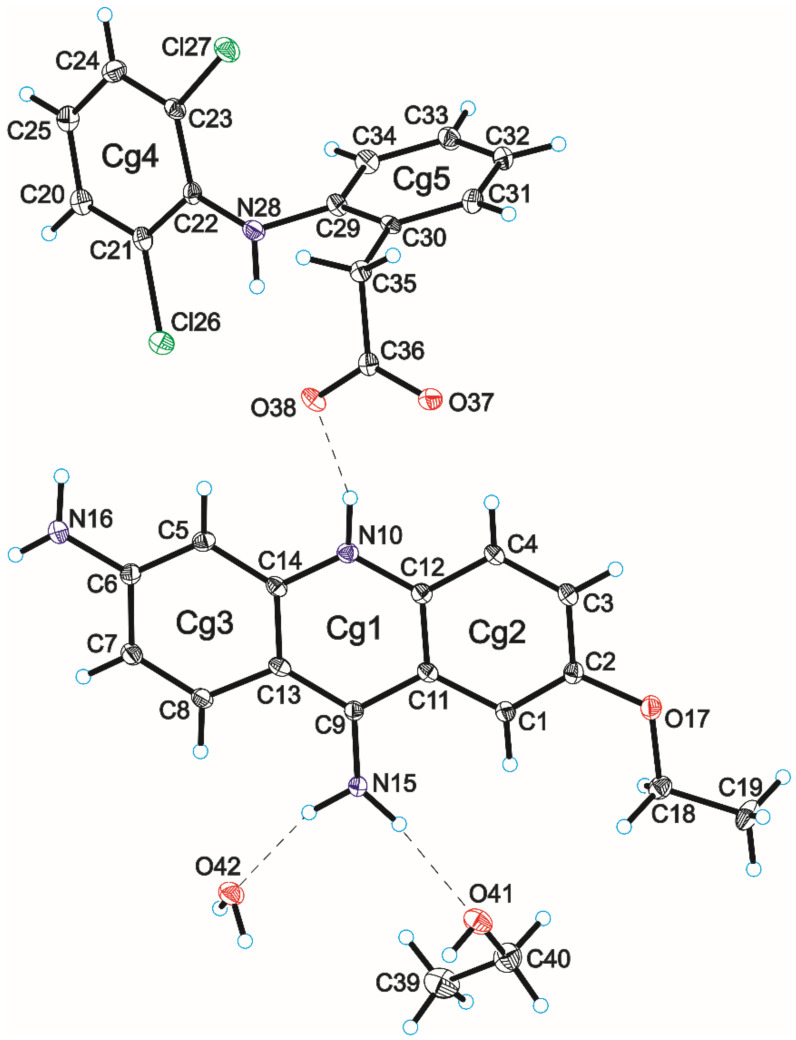
Molecular structure of compound **2** showing the atom-labelling scheme (Cg1, Cg2, Cg3, Cg4, and Cg5 denote the ring centroids; hydrogen bonds are represented by dashed lines).

The single-crystal X-ray diffraction measurements show that compound **2** crystallizes in the triclinic *P*-1 space group with one 6,9-diamino-2-ethoxyacridinium cation, one diclofenac anion, one ethanol molecule and one water molecule in the asymmetric unit (Figure 4).

**Figure 5:**
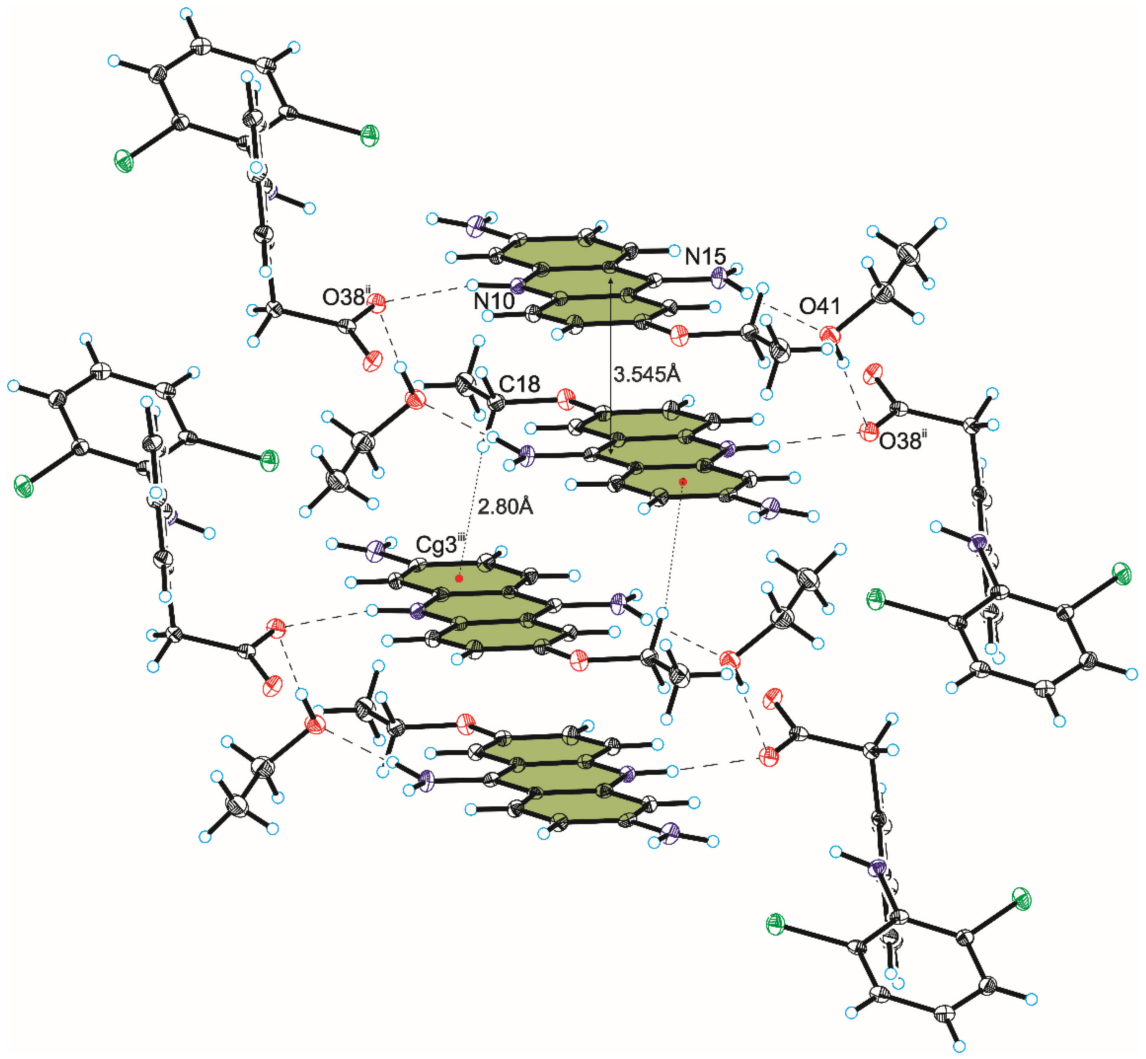
Heterohexamer formed by ethoxyacridinium and diclofenac ions and ethanol molecule in compound **2** (hydrogen bonds are represented by dashed lines, whereas C–H⋯π interactions are represented by dotted lines).

The lengths of C–O bonds from carboxylic group ranging from 1.248 Å to 1.255 Å indicating a proton transfer occurring between the carboxylic group of diclofenac and 6,9-diamino-2-ethoxyacridine. An ethacridine skeleton is nearly planar, while in the diclofenac anion the angle between the planes of aromatic rings is 71.6°, and the angle –C–N–C– is 122°. In the diclofenac anion, there is an intramolecular N–H⋯O hydrogen bond [*d*(H28⋯O38) = 2.43(6) Å, and ∠(N28–H28⋯O38) = 141(6)°]. In the crystal of compound **2**, the 6,9-diamino-2-ethoxyacridine cation is linked with the diclofenac anion by N_(acridine)_–H⋯O_(carboxy)_ hydrogen bond [*d*(H10⋯O38) = 2.01(6) Å and ∠(N10–H10⋯O38) = 157(6)°], and with the ethanol molecule by N_(9-amino)__–_H⋯O_(ethanol)_ hydrogen bond [*d*(H15B⋯O41) = 2.00(6) Å and ∠(N15–H15B⋯O41) = 173(6)°], while the ethanol molecule interact with the diclofenac anion via O_(ethanol)__–_H⋯O_(carboxy)_ hydrogen bond [*d*(H41⋯O38) = 2.04 Å and ∠(O41–H41⋯O38) = 176°], and form a cyclic heterohexamer (Table 2, Figure 5). This heterohexamer is stabilized by the weak C_(acridine)_–H⋯O_(carboxy)_ hydrogen bonds between the C-4 atom of the acridinium cation and the O-atom from the carboxylate group of the diclofenac anion [*d*(H4⋯O37) = 2.59 Å, and ∠(C4–H4⋯O37) = 151°] and N_(6-amino)_–H⋯Cl hydrogen bond between the amino group and a Cl-atom from the diclofenac anion [*d*(H16A⋯Cl26) = 2.91(9) Å, and ∠(N16–H16A⋯Cl26) = 133(7)°] (Table 2, Figure 5).

**Figure 6:**
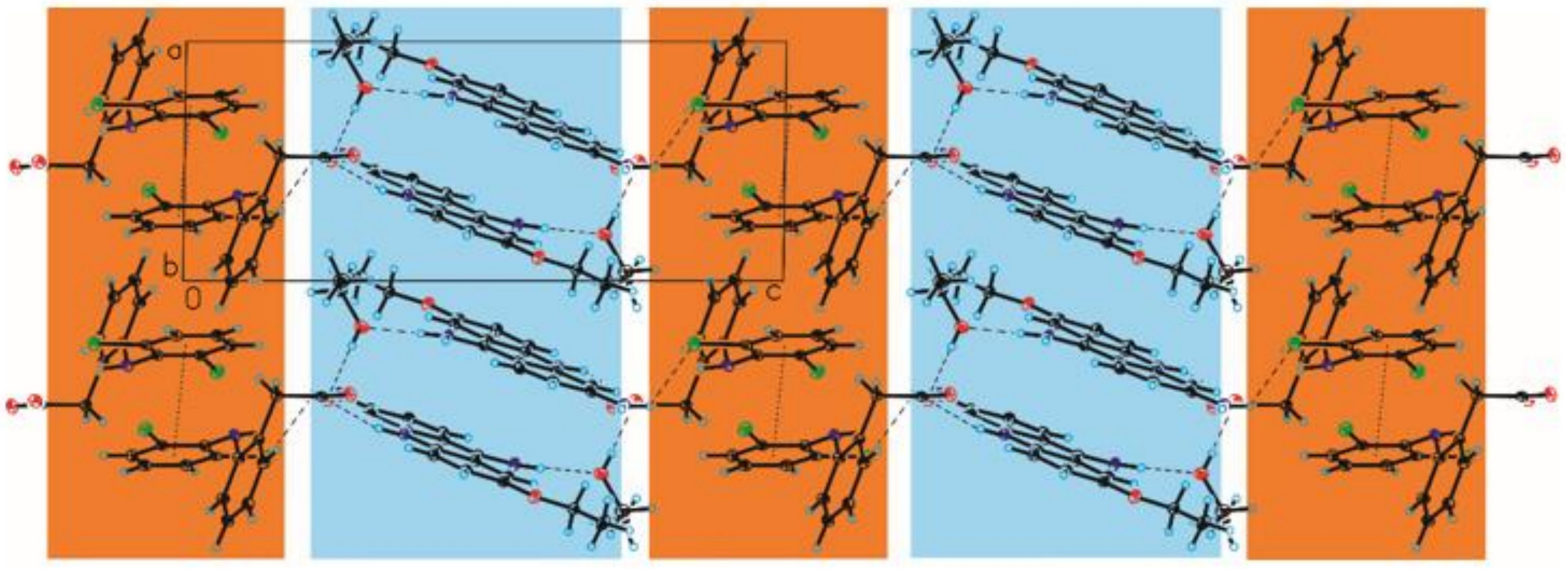
Crystal packing of compound **2** viewed along the *b*-axis (hydrogen bonds are represented by dashed lines, whereas π–π interactions are represented by dotted lines).

Adjacent 6,9-diamino-2-ethoxyacridine cations engaged in the formation of heterohexamer interact with each other via π–π interactions with the distance between centroids ranging from 3.835 (3) Å to 3.917 (4) Å and separation 3.545 Å between the mean planes of the 6,9-diamino-2-ethoxyacridine skeleton (Table 4, Figure 5). Moreover, the adjacent heterohexamers are linked by C–H⋯π interaction [*d*(H18A⋯Cg3) = 2.80 Å, and ∠(C18–H18⋯Cg3) = 150°] to form blocks (Table 3, Figure 6—highlighted in blue). In turn, the adjacent diclofenac anions interact via π–π intermolecular interaction with distance between centroids 3.553 (4) Å and separation 3.432 Å, to form columns along the *a*-axis (Table 4, Figure 6—highlighted in orange), which connect adjacent blocks of acridines.

The adjacent blocks are also connected through a water molecule by N_(9-amino)_–H⋯O_(water)_ hydrogen bond [*d*(H15A⋯O42) = 1.91 (8) Å, and ∠(N15–H15A⋯O42) = 163 (7)°] between the amino group of the acridinium cation and water molecule, the O_(water)_–H⋯O_(ethoxy)_ hydrogen bond [*d*(H42B⋯O17) = 2.17 (7) Å, and ∠(O42–H42B⋯O17) = 154 (6)°] between the water molecule and ethoxy group from the acridinium cation, the O_(water)_–H⋯O_(carboxy)_ hydrogen bond [*d*(H42A⋯O37) = 1.95 (6) Å, and ∠(O42–H42A⋯O37) = 158 (8)°] between the water molecule and an O-atom from the carboxylate group of the diclofenac anion and the weak C_(acridine)_–H⋯O_(water)_ hydrogen bonds between the C-8 atom of the acridinium cation and the water molecule [*d*(H8⋯O42) = 2.45 Å, and ∠(C8–H8⋯O42) = 165°]. Alternately arranged blocks and columns are connected by N_(6-amino)_–H⋯O_(carboxy)_ hydrogen bond [*d*(H16B⋯O37) = 2.19 (7) Å, and ∠(N16–H16B⋯O37) = 171 (7)°], to form a 3D framework structure (Figure 6).

## 4. Discussion

The title compounds crystallize in the triclinic *P*-1 space group, but they are not isostructural. Compound **1** crystallizes as salt cocrystal with one acridinium cation, one diclofenac anion and one diclofenac molecule in the asymmetric unit. In CSD, there are only five structures of salt cocrystal (REFCODES: XUVYAP01, WABHOA, WABHUG, WABJAO, XUVQEL) [49,52,77]. In the structures deposited in the CSD, the angle between the planes of aromatic rings is from 59.6° to 78.3° and the angle –C–N–C– is in the range 116.3–125.2° in diclofenac molecule which corresponds to the angle between the plane of aromatic rings (60.6°) and angle –C–N–C– (123.5°) observed in the diclofenac molecule in the crystal of compound **1**. Simultaneously, in the structures deposited in the CSD-containing diclofenac anion, the angle between the planes of aromatic rings is from 58.2° to 80.4° and the angles –C–N–C– is in the range 120.3° to 125.2°, while the angles between the plane of aromatic rings is 87.2° and the angles –C–N–C– is 118.9° in compound **1**. In this crystal structure, we observed specific cyclic heterotetramer *bis*[⋯cation⋯anion⋯], containing N_(acridine)_–H⋯O_(diclofenac)_ and C_(acridine)_–H⋯π_(diclofenac)_ intermolecular interactions, which is not observed in the crystal structures in our previous studies (about acridine) [64]. Additionally, acridinium cations do not form π–stacked columns, whereas the neighboring heterotetramers are connected by columns made of diclofenac molecules, which interact by C–Cl⋯π intermolecular interaction with each other. Compound **2** was obtained as an ethanol solvate monohydrate salt, and crystallizes with one acridinium cation, one diclofenac anion, one ethanol molecule and one water molecule in the asymmetric unit. Moreover, there is only one structure containing a diclofenac anion and an ethanol molecule, and there are not any structures containing an ethanol molecule and a water molecule in CSD. In the crystal of **2**, the angle between the plane of aromatic rings is 71.6° and the angle –C–N–C– is 122° in the diclofenac anion, which corresponds to the data obtained from CSD. It means that the angles between the plane of aromatic rings is bigger in compound 1, but the angle –C–N–C– is smaller than observed in the structure of compound 2. In this crystal structure we observed cyclic heterohexamer *bis*[⋯cation⋯ethanol⋯anion⋯]. The similar heterohexamer we observed in the crystal structures of ethacridinium phthalate isobutanol solvate [66], where instead of the ethanol molecule there is an isobutanol molecule, but the distance between mean planes of ethacridinium is longer in the crystal of compound **2** (3.431 Å and 3.545 Å in the crystal of ethacridinium phthalate isobutanol solvate and compound **2**, respectively). In the crystal structure of compound 2, neighboring heterohexamers are linked via π–π interactions and π–stacked columns of ethacridine are formed, whereas inversely oriented diclofenac anions interact through π–π interactions each other and linked these columns. The differences in the crystal packing of the title compounds influence the Kitaigorodskii type of packing index —the percentage of filled space are equal to 67.9% and 68.5% for compound 1 and 2, respectively (the Kitaigorodskii type of packing index).

## 5. Conclusions

Structural investigations of compounds obtained from the crystallization of diclofenac with acridines show that salt cocrystal (**1**) or solvate monohydrate salt (**2**) are formed. The title compounds crystallize in the triclinic *P*-1 space group, but they are not isostructural. Moreover, the crystal packing of both compounds is differed. In the crystals of title compounds, diclofenac and acridines ions and solvent molecules interact via N–H⋯O, O–H⋯O, and C–H⋯O hydrogen bonds, as well as C–H⋯π and π–π interactions, and form heterotetramer *bis*[⋯cation⋯anion⋯] or heterohexamer *bis*[⋯cation⋯ethanol⋯anion⋯] in compound **1** and **2**, respectively. In the crystal of compound **1**, the acridine cations and the diclofenac anions interact via N–H⋯O hydrogen bond, C–H⋯π and π–π interactions to form blocks, whereas the diclofenac molecules interact via C–Cl⋯π interactions to form columns. In the crystal of compound **2**, the ethacridine cations interact via C–H⋯π and π–π interactions building blocks, while diclofenac anions interact via π–π interactions to form columns. The results of our research provide new insight into the unique crystal structure containing diclofenac and acridine/6,9-diamino-2-ethoxyacridine (ethacridine) and can be used to obtain and design new forms of drugs involving these APIs.

## Figures and Tables

**Table 1:** Crystal data and structure refinement for compounds **1** and **2**.

Compound	1	2
Chemical formula	C_41_H_31_Cl_4_N_3_O_4_	C_31_H_34_Cl_2_N_4_O_5_
Formula weight/g·mol^−1^	771.49	613.52
Crystal system	triclinic	triclinic
Space group	*P*-1	*P*-1
a/Å	10.1582(4)	7.6339(10)
b/Å	12.3057(6)	10.2331(17)
c/Å	14.8978(7)	19.250(3)
α/°	91.050(4)	84.860(12)
β/°	99.988(3)	89.009(11)
γ/°	99.111(4)	88.337(13)
V/Å^3^	1808.9(1)	1497.0(4)
Z	2	2
T/K	295(2)	295(2)
λMo/Å	0.71073	0.71073
ρ_calc_/g·cm^−3^	1.416	1.361
F(000)	796	644
µ/mm^−1^	0.375	0.264
θ range/°	3.31–25.00	3.29–25.00
Completeness θ/%	99.7	99.8
Reflections collected	12784	9800
Reflections	6353	5246
unique	[R_int_ = 0.0297]	[R_int_ = 0.0883]
Data/restraints/parameters	6353/0/485	5246/3/411
Goodness of fit on *F*^2^	1.021	1.007
Final R_1_ value (I > 2σ(I))	0.0445	0.0891
Final wR_2_ value (I > 2σ(I))	0.0844	0.1855
Final R_1_ value (all data)	0.0703	0.2190
Final wR_2_ value (all data)	0.0945	0.2559
CCDC number	2128054	2128053

**Table 2:** Hydrogen bonds geometry for compounds **1** and **2**.

Compound	D–H⋯A	*d*(D–H) [Å]	*d*(H⋯A) [Å]	*d*(D⋯A) [Å]	∠D–H⋯A [°]
	N23–H23⋯O33	0.81 (2)	2.39 (2)	2.977 (2)	130 (2)
	N42–H42⋯O52	0.82 (2)	2.36 (2)	3.044 (3)	142 (2)
	N10–H10⋯O33	0.87 (2)	1.79 (2)	2.648 (2)	170 (2)
**1**	O51–H51⋯O32	0.90 (4)	1.69 (3)	2.560 (3)	164 (3)
	C45–H45⋯O32 ^i^	0.93	2.71 (1)	3.603	160 (1)
	C4–H4⋯O32	0.93	2.40	3.292 (3)	160
	C8–H8⋯O52 ^ii^	0.93	2.50	3.342 (3)	150
Symmetry code: ^(i)^ 1 − x, −y, 1 − z; ^(ii)^ 1 − x, 1 − y, 1 − z.					
	N28–H28⋯O38	0.91 (6)	2.43 (6)	3.188 (7)	141 (6)
	N10–H10⋯O38	0.94 (6)	2.01 (6)	2.898 (7)	157 (6)
**2**	N15–H15B⋯O41	0.95 (6)	2.00 (6)	2.945 (8)	173 (6)
	N15–H15A⋯O42	0.95 (8)	1.91 (8)	2.832 (9)	163 (7)
	N16–H16B⋯O37 ^i^	0.84 (7)	2.19 (7)	3.015 (1)	171 (7)
	N16–H16A⋯Cl26	0.93	2.91 (9)	3.603	133 (7)
	O41–H41⋯O38 ^ii^	0.82	2.04	2.864 (7)	176
	O42–H42A⋯O37 ^ii^	0.85 (4)	1.95 (6)	2.753 (6)	158 (8)
	O42–H42B⋯O17 ^i^	0.83 (7)	2.17 (7)	2.941 (7)	154 (6)
	C4–H4⋯O37	0.93	2.59	3.438 (8)	151
	C8–H8⋯O42	0.93	2.45	3.353 (9)	165
Symmetry code: ^(i)^ x, 1 + y, z; ^(ii)^ 1 − x, 1 − y, 1 − z.					

**Table 3:** C–X⋯π interactions geometry for compounds **1** and **2** (X = H/Cl).

Compound	C–X⋯Cg	*d*(X⋯Cg) [Å]	*d*(C⋯Cg) [Å]	∠C–X⋯Cg [°]
	C1–H1⋯Cg4 ^ii^	2.79	3.697 (3)	165
	C7–H7⋯Cg7 ^ii^	2.90	3.769 (3)	156
**1**	C16–Cl21⋯Cg7 ^iii^	3.885 (1)	5.138 (2)	127.92 (8)
	C35–Cl40⋯Cg6 ^iv^	3.725 (1)	4.267 (2)	95.89 (8)
Symmetry code: ^(ii)^ 1 − x, 1 − y, 1 − z; ^(iii)^ 1 + x, 1 + y, z; ^(iv)^ 1 − x, −y, 2 − z.				
**2**	C18–H18A⋯Cg3 ^iii^	2.80	3.666 (7)	150
Symmetry code: ^(iii)^ 2 − x, 1 − y, 1 − z.				

**Table 4:** π–π interactions geometry for compounds **1** and **2**.

Compound	CgI ^a^	CgJ ^a^	CgI⋯CgJ ^b^ [Å]	Dihedral Angle ^c^ [°]	Interplanar Distance ^d^ [Å]	Offset ^e^ [Å]
	1	1 ^ii^	3.737 (1)	0.0 (1)	3.409 (9)	1.533
	1	2 ^ii^	3.841 (1)	2.2 (1)	3.404 (9)	1.672
**1**	2	3 ^ii^	3.796 (2)	3.8 (1)	3.464 (1)	1.388
	1	4 ^v^	3.662 (1)	3.9 (1)	3.345 (9)	1.293
	3	4 ^v^	3.917 (1)	2.4 (1)	3.459 (1)	1.883
Symmetry code: ^(ii)^ 1 − x, 1 − y, 1 − z; ^(v)^ 2 − x, 1 − y, 1 − z.						
	1	1 ^ii^	3.835 (3)	0.0 (3)	3.517 (2)	1.528
**2**	2	3 ^ii^	3.917 (4)	2.1 (3)	3.530 (3)	1.596
	4	4 ^iv^	3.553 (4)	0.0 (3)	3.432 (3)	0.919
Symmetry code: ^(ii)^ 1 − x, 1 − y, 1 − z; ^(iv)^ 1 − x, 1 − y, 2 − z.						

^(a)^ Cg represents the center of gravity of the rings. ^(b)^ Cg⋯Cg is the distance between ring centroids. ^(c)^ The dihedral angle is that between the mean planes of CgI and CgJ. ^(d)^ The interplanar distance is the perpendicular distance from CgI to ring J. ^(e)^ The offset is the perpendicular distance from ring I to ring J.

## Data Availability

Data are contained within the article.
